# Transcriptome and metabonomics combined analysis revealed the energy supply mechanism involved in fruiting body initiation in Chinese cordyceps

**DOI:** 10.1038/s41598-023-36261-7

**Published:** 2023-06-12

**Authors:** Li He, Fang Xie, Gang Zhou, Zhao He Chen, Jing Yi Wang, Cheng Gang Wang

**Affiliations:** School of Biological and Pharmaceutical Engineering, Lanzhou Jiaotong University, Lanzhou, Gansu People’s Republic of China

**Keywords:** Microbiology, Medical research

## Abstract

Chinese cordyceps was one of most valuable traditional Chinese medicine fungi. To elucidate the molecular mechanisms related to energy supply mechanism involved in the initiation and formation of primordium in Chinese cordyceps, we performed the integrated metabolomic and transcriptomic analyses of it at pre-primordium period, primordium germination period and after-primordium period, respectively. Transcriptome analysis showed that many genes related to ‘starch and sucrose metabolism’, ‘fructose and mannose metabolism’, ‘linoleic acid metabolism’, ‘fatty acids degradation’ and ‘glycerophospholipid metabolism’ were highly up-regulated at primordium germination period. Metabolomic analysis showed many metabolites regulated by these genes in these metabolism pathways were also markedly accumulated at this period. Consequently, we inferred that carbohydrate metabolism and β-oxidation pathway of palmitic acid and linoleic acid worked cooperatively to generate enough acyl-CoA, and then entered TCA cycle to provide energy for fruiting body initiation. Overall, our finding provided important information for further exploring the energy metabolic mechanisms of realizing the industrialization of Chinese cordyceps artificial cultivation.

## Introduction

The Chinese cordyceps, one of the precious traditional herbal medicines in China, is a complex of batmoth larvae with *Ophiocordyceps* *sinensis* (*O.* *sinensis*), used for health care and cure diseases and even other medical effects^1^. It is extremely expensive for its medicinal benefits and decreasing supply. But it has been on the verge of extinction due to its low-recovery with complicated and severe habitations^2^. In addition, long-term over-exploitation had shrunk its yield, which has seriously threatened the sustainable utilization of resources^3^. In order to solve the problems of market shortage and maintain the natural ecological environment of this natural resource, the research regarding its artificial cultivation technology has been arousing extensive attention. Although some breakthroughs have been made in its artificial cultivation, the internal mechanism of morphological changes during its growth was still unclear^4,5^.

It was a complicated process for Chinese cordyceps to initiate fruiting body, which was closely related to the regulation of specific expression genes at different growth stages. Encouragingly, Xiong et al. reported for the first time that cDNA library of Chinese cordyceps at all growth stages was constructed, which laid a foundation for the study of fruiting body development of entomogenous fungi^6^. Based on this cDNA library, Zheng et al. found that deletion of the active factor gene(*cvn*) could inhibit the development of its fruiting body^7^. Subsequently, our team found that *VeA* and *Hsp70* genes were specifically expressed during the fruiting body initiation of Cordyceps cordyceps^8^. As outlined recently, Chinese cordyceps contained six *MYB* transcription factors, among which expression of *MYB-3* was the highest at the maturation stage of its fruiting body, suggesting that *MYB-3* might be involved in its sexual development^1^. Although so much efforts have been made to explore regulatory genes related to fruiting body initiation, the key gene for triggering this process was still unknown.

At particular stage in the life cycle of Chinese cordyceps, it can synthesis some compatible osmolytes, including sugars, amino acids, and secondary metabolites. These metabolites were imperative to some stages of reproduction. In fungi field, proline content in mushroom cap was significantly higher than that in stalk, which might play an important role in promoting the development of mushroom cap^9^. In *Cordyceps* *cicadae*, Qu et al. found that nicotinamide and gibberellin were linked with its reproductive growth and spore maturation^10^. It was reported that arginine synthesis was involved in the development, conidia, appressorium formation and morphogenesis in oryzae^11^. Meanwhile, betaine played an important role in the regulation of stress resistance of fungi^12^. Compared to large fungus or *Cordyceps* *militaris*, far less was known about initiation of primordium, even though there was literature related to differential compounds were identified in its head^13^, further exploration was unmentioned. The fruiting body of Chinese cordyceps only initiated at its head, whether this unique morphological feature was related to the presence of certain metabolites in the moth's head was an unsolved mysteries.

Enhanced energy metabolism could provide momentum for the morphological development and maturation of fruiting bodies in many edible-medicinal fungi^14^. Some findings have deemed that upregulation of proteins related energy production were described in the fruiting body initiation stage in fungus^15,16^. In *Dictyophora* *indusiate* (*D.* *indusiata*), proteins involved in the glycolysis/gluconeogenesis pathway, which could produce acetyl-CoA by progressive decomposition of glucose to provide substrate to the TCA cycle in fruiting body initiation^17^. As outlined recently, combined proteomic and metabolomic analysis showed that glycerol phospholipids were hydrolyzed significantly during the fruiting body initiation, and the protein was mainly catabolic. Along with vigorous metabolism, energy production was enhanced by upregulated TCA cycling and oxidative phosphorylation in *D.* *indusiata*^18,19^. However, it was still unknown about energy supply mechanism at the initiation of fruiting body stage in Chinese cordyceps.

Next generation sequencing and more specifically RNA-Sequencing (RNA-Seq), have became popular and comprehensively informative approach to predict and validate novel key regulators and their direct and indirect targets in fungi signaling networks^20^. Metabolomics could aid the discovery of fungi metabolites related to key nodes for growth and development^21^. Combined transcriptome and metabolome could allow quantitative mapping of transcripts directly to metabolic pathways involved in initiating fruiting body. Primordium germination was the key growth node of Chinese cordyceps from mycelium growth to fruiting body. It was essential to understand the metabolic changes, transcriptional regulation, and physiological responses of bioactive and signaling compounds when fruiting body initiated. Thus, in this study, we combined metabolomic and transcriptome analysis to identify the energy supply pathways and related different expression genes (DEGs) and metabolites (DEMs) involved in fruiting body initiation. The aim was to identify the key DEGs and DEMs related energy supply at the initiation of fruiting body stage, which will lay the foundation for the intrinsic mechanism of artificial cultivation for Chinese cordyceps.

## Materials and methods

### Materials

Materials S1 (pre-primordium) batmoth larva inoculated with *Ophiocordyceps* *sinensis* (*O.* *sinensis*) were provided by Hangzhou Mingxu Biotechnology Co., LTD. Materials were cultured as described by Zhao et al. with some changes^1^. When the Chinese cordyceps reached at the beginning of germination stage (about 30 days), they were randomly and equally divided into two groups: one group collected as materials (primordium germination), another group continued to culture until primordium formated (55 days), these samples as materials (after-primordium), 100 mg was taken from each of the 3 samples at different stages, and 3 biological replicates were taken from each group, all samples were immediately frozen in liquid nitrogen and stored at − 80 °C.

### Observation of stroma germination by scanning electron microscopy

Scanning electron microscope was used to observe three stages of head materials. Samples were fixed in 2.5% glutaraldehyde at 4 °C for 12 h, then washed by 0.1 mol L^−1^ phosphoric acid buffer for 15 min with three times. After dehydration by gradient ethanol (30%, 50%, 70%, 90%, 95%) for one time, followed dehydrated with 100% ethanol at twice. Finally, iso-amyl acetate was used to treat it for 2 h and then put it in freeze dryer for 15 h. The shape and size of mycelium were observed and measured under scanning electron microscope after it was sprayed gold.

### Polyphenols, polysaccharides and mannitol content determination

Polyphenols contents was determined according to folin-phenol colorimetric method^22^. Polysaccharide content was determined by anthrone-sulfuric acid method^23^. Mannitol content was determined by sodium periodate colorimetry^24^. Each experiment was repeated at least three replicates.

### Total RNA extraction, library construction, quality inspection and sequencing

RNA extraction, quantification, and transcriptome sequencing were conducted on the basis of our previous method^24^. Library construction was completed by Shanghai Oui Biomedical Technology Co. LTD. The library quality was assessed on the Agilent Bioanalyzer 2100 system, and paired-end reads were generated. High quality clean reads obtained by filtering raw reads with Trimmomatic software^25^, which was blasted to caterpillar fungus reference gene (https://ftp.ncbi.nlm.nih.gov/genomes/all/GCA/012/934/285/GCA_0129342) using hisat2 software^26^. DESeq was used to analyze the difference of gene expression levels in different samples^27^. We used DIAMOND-BLASTX v0.8.24 to compare the assembled transcript sequences using KEGG (http://www.kegg.jp/kegg/kegg1.html), GO, Swiss-Prot, KOG, and NR databases^28^. The nbinom test was used to calculate the *p* value and foldchange values for the difference comparison. Differential genes with *p* value < 0.05 and FC value > 2 were selected for GO and KEGG enrichment analysis^26^. Heat maps were used to show the expression patterns of different genes in different samples with TBtools software^28^.

### Real time PCR analysis

The RNA sample used for transcriptome sequencing as the material, cDNA was synthesized by reverse transcription using qPCR kit (Vazyme), and qRT-PCR analysis was performed using LightCycler480II type fluorescence quantitative PCR instrument. The reference gene was *18SrRNA* from *Trichoderma* *chinensis*. Relative gene expression was calculated by 2^−∆∆Ct^ method. Specific primer information was shown in Table 1.

**Table 1:** PCR primer used in this study.

Gene symbol	Forward primer	Reverse primer
radR	AAAGTCTTGGGCCTTTGC	GGTATTTGTCCGCCAAACTG
65E11.140	TGGTCGAACTCCGAGGACAA	GTTTCAGCTATATGAACCCAGT
T16L24.250	GATCTGTCCGGCTGGATT	TGCAACATCGCGGAGAAC
CYP205	TATCAAGACTCCAGGCAAGG	CTGATAGTTGCTTGACACAG
tyr1	TGCGTGGTGGAGCATGAA	ATCCACAACGGAATACACTG
YCR10C	TGCAGAGCAGGCTTACAC	CTTGAGAGACCATGGACTGA
YHR009C	ATGGAGCGCAACATTGTC	GGGTTGAATTTGGGATGGC
ATEG_00912	CGAGATCCAACGGAATCAC	TCATGTAGCGCAGATGCAC
CaO19.10303	CTTTACCCAGCTTGTCTGGA	ACACAACAATGGTACGCC
SPBC29A10.02	TCGCTCTGCCTACACAAC	TTGAAATGGCCGACTTGG
*Ophiocordyceps* *sinensis* *18S*	GCAGTGGCATCTCTCAGTC	TCATCGATGCCAGAACC

### Metabolite extraction procedure

Metabolite extraction by Wang et al. with some changes^19^. 500 mg accurately weighed sample was transferred to a 1.5 mL Eppendorf tube. 20 μL of l-2-chlorophenylalanine (0.6 mg/mL) dissolved in methanol as internal standard and 400 μL mixture of methanol and water (4/1, v/v) were added to each sample, two small steel balls were added to the tube and samples were placed at − 20 °C for 2 min. Then grinded at 60 Hz for 2 min, and the whole samples were extracted by ultrasonic for 30 min in ice-water bath, then placed at − 20 °C for 20 min. Samples were centrifuged at 4 °C (13,000 rpm) for 10 min, 300 μL supernatants was put into LC–MS sample vial and dried. Redissolved with 300 μL methanol–water (V:V = 1:4), placed at − 20 °C for 20 min. After centrifugated for 10 min (13,000 rpm at 4 °C), 150 μL supernatant was absorbed with a syringe and filtered with a 0.22 μm organic phase pinhole filter. The supernatant was transferred to LC sample vial and stored at − 80 °C until LC–MS analysis. QC were prepared by mixing aliquot of the all samples to be a pooled sample.

### Chromatography analysis

Chromatography analysis by Wang et al. with some changes^19^_._ A Dionex Ultimate 3000 RS UHPLC fitted with Q-Exactive plus quadrupole-Orbitrap mass spectrometer equipped with heated electrospray ionization (ESI) source (Thermo Fisher Scientific, Waltham, MA, USA) was used to analyze the metabolic profiling in both ESI positive and ESI negative ion modes. An ACQUITY UPLC HSS T3 column (1.8 μm, 2.1 × 100 mm) were employed in both positive and negative modes. The binary gradient elution system consisted of (A)water (containing 0.1% formic acid, v/v) and (B) acetonitrile (containing 0.1% formic acid, v/v) and separation was achieved using the following gradient: 0 min, 5% B; 2 min, 5% B; 4 min, 25% B; 8 min, 50% B; 10 min, 80% B; 14 min,100% B; 15 min, 100% B; 15.1 min, 5% and 16 min, 5% B. The flow rate was 0.35 mL/min and column temperature was 45 °C. All the samples were kept at 4 °C during the analysis. The mass range was from m/z 100 to 1000. The resolution was set at 70,000 for the full MS scans and 17,500 for HCD MS/MS scans. The collision energy was set at 10, 20 and 40 eV. The mass spectrometer operated as follows: sprayvoltage, 3800 V (+) and 3000 V (−); sheath gas flow rate, 35 arbitrary units; auxiliary gas flow rate, 8 arbitrary units; capillary temperature, 320 °C; Aux gas heater temperature, 350 °C; S-lens RF level, 50. The QCs were injected at regular intervals throughout the analytical run to provide a set of data from which repeatability can be assessed.

### Metabolite qualitative and quantitative analysis

Metabolite extraction and profiling was completed by Shanghai Oui Biomedical Technology Co., LTD. Progenesis QI V 2.3 software was used to process the raw data. The Human Metabolome Database (HMDB), Lipidmaps (V2.3), METLIN databases were used to identify compounds. The data matrix was composed of positive and negative ion data. Principal component analysis (PCA) was carried out by introducing matrix into R to observe the stability of the whole distribution between samples and the whole analysis process. Orthogonal partial least squares discriminant analysis (OPLS-DA) and partial least squares discriminant analysis (PLS-DA) were used to distinguish the differences between metabolite groups. The variable weight value (VIP) can be used to measure the influence intensity and explanatory ability of the expression pattern of each metabolite on the classification and discrimination of each group. Differential metabolites with VIP value > 1 and *p* value < 0.05 were screened, and metabolic pathway enrichment analysis of it was performed using KEGG database.

### Combined transcriptome and metabolome analyses

We carried out cojoint analysis on the DEGs and DEMs to explore the degree of enrichment of pathways. Gene-metabolite networks with a Pearson correlation coefficient (PCC) > 0.8 were used to construct the transcript-metabolite network^29^.

## Results

### Changes in fruiting body formation appearance and physiological indicators before and after fruiting body initiation

**Figure 1:**
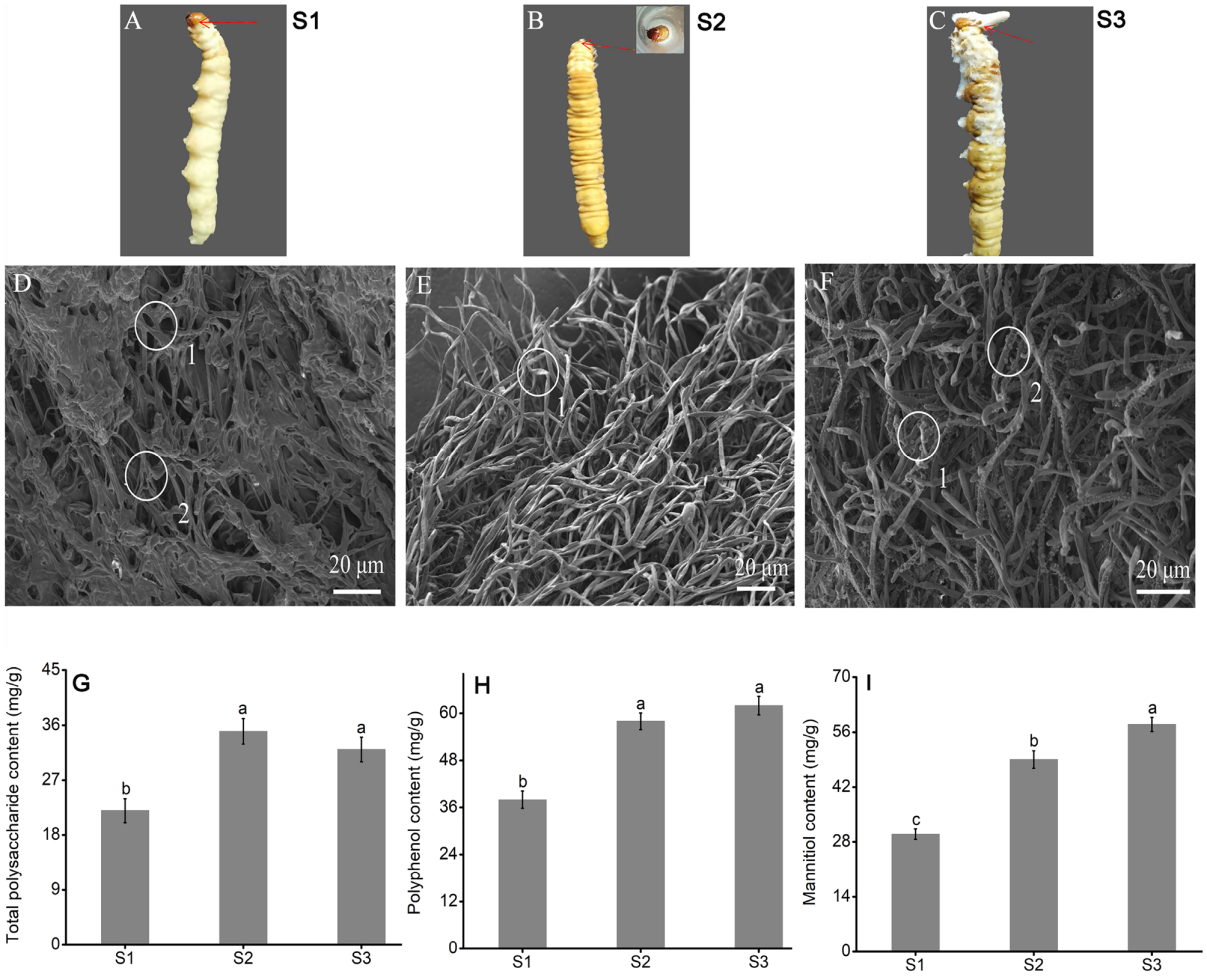
Sample collections of Chinese cordyceps for RNA-Seq. (**A)** Pre-primordium. (**B**) primordium. (**C**) After-primordium. (**D**) SEM of the pre-primordium mycelium (worm). (**E**) SEM of the primordium (the mycelium broke through the head). (**F**) SEM of the after primordium (the formation of stroma). (**A**–**C**) (Red shears) represented sampling site, respectively. (**D**) (1, 2), fusion of mycelium; (**E**) (1), slender and curved mycelium; (**F**) (1, 2), microscopic particles. Bar was 20 mm. (**G**) Total polysaccharide contents. (**H**) Polyphenol content. (**I**) Mannitol content. Bars represent mean ± SE (n = 3) and different lower-case letters represent significant difference at *p* < 0.05.

The appearance of fruiting body formation was shown in Fig. 1, it experienced pre-primordium period (S1) (Fig. 1A), primordium germination period (S2) (Fig. 1B) and after-primordium period (S3) (Fig. 1C). In S1 period, mycelium was stubby, cross-linking and winding with low curvature, its surface was verrucous with visible fold (Fig. 1D). In S2 period, the mycelium broken through the head of worm and formed a needle-like primordium, mycelium was slender, high curvature, loose arrangement and its surface was smooth (Fig. 1E). Subsequently, the primordium further initiated to grow, the mycelium thickness of which was evenly and tightly arranged (Fig. 1F), suggesting morphological characteristics of three stages mycelium had great differences. The changes in total polysaccharides, polyphenol and mannitol content of Chinese cordyceps were shown in Fig. 1G–I. The total polysaccharides was remarkably accumulated at S2 period and then showed downward trend, while polyphenol and mannitol content showed continuously increasing trend with fruiting body development. The accumulation of these metabolites might be closely related to the initiation of fruiting bodies.

### UPLC–MS/MS-based quantitative metabolic analyses of Chinese cordyceps mycelium related to fruiting body initiation

**Figure 2:**
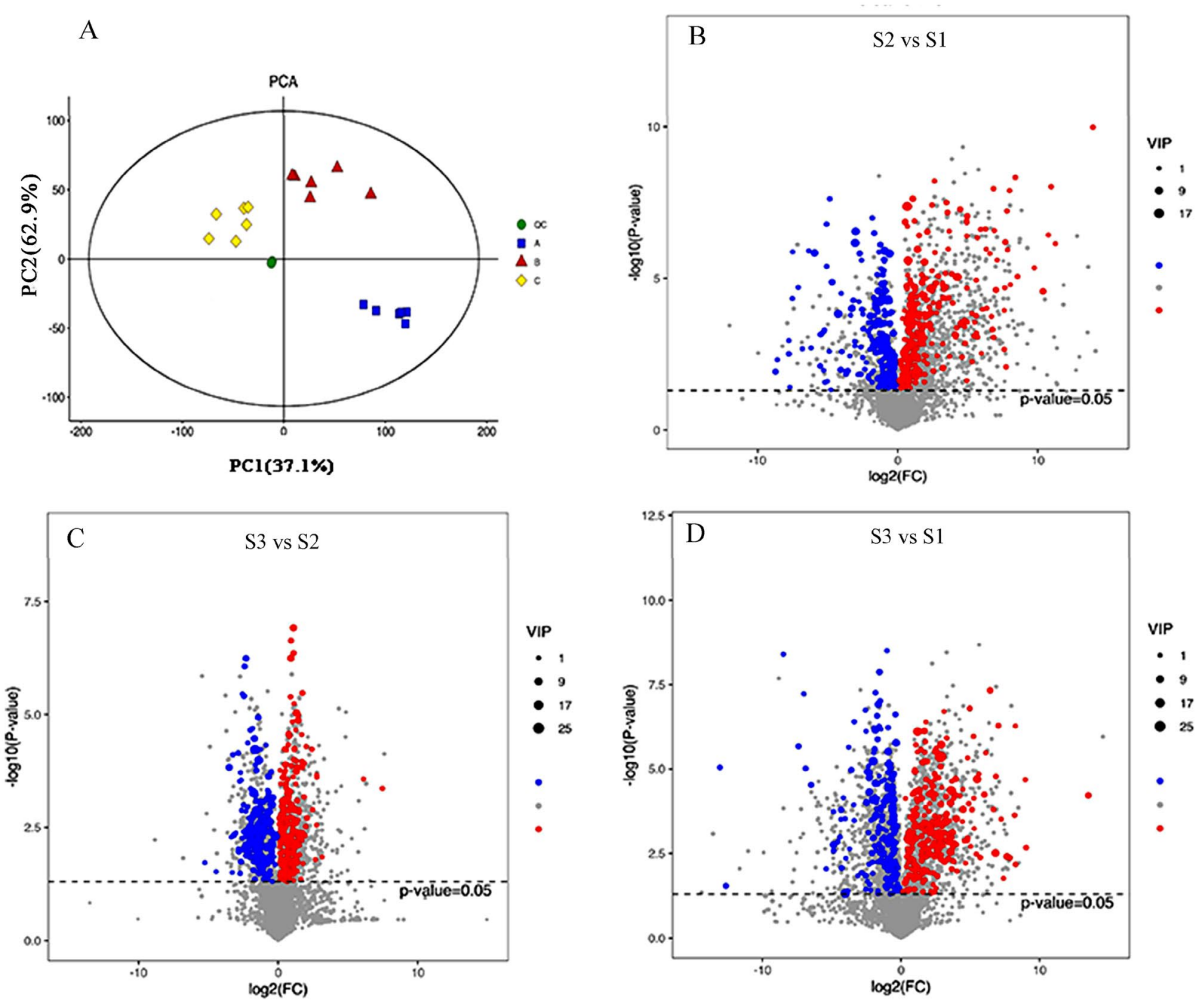
Differentially accumulated metabolites between three comparable groups. (**A**) Principal component analysis (PCA) of the variance-stabilized estimated raw counts of differentially accumulated metabolites. Bar plot showing numbers of DEMs in S2 vs S1 (**B**), S3 vs S2 (**C**), and S3 vs S1 (**D**) groups. The horizontal axis represents the differential expression multiple, and the vertical axis represents the degree of difference in metabolites meaning. The red dots indicated the upregulated expressed metabolites (Log_2_ FC ≥ 2), the blue dots indicated the downregulated expressed metabolites (Log_2_ FC ≤ 0.5).

**Figure 3:**
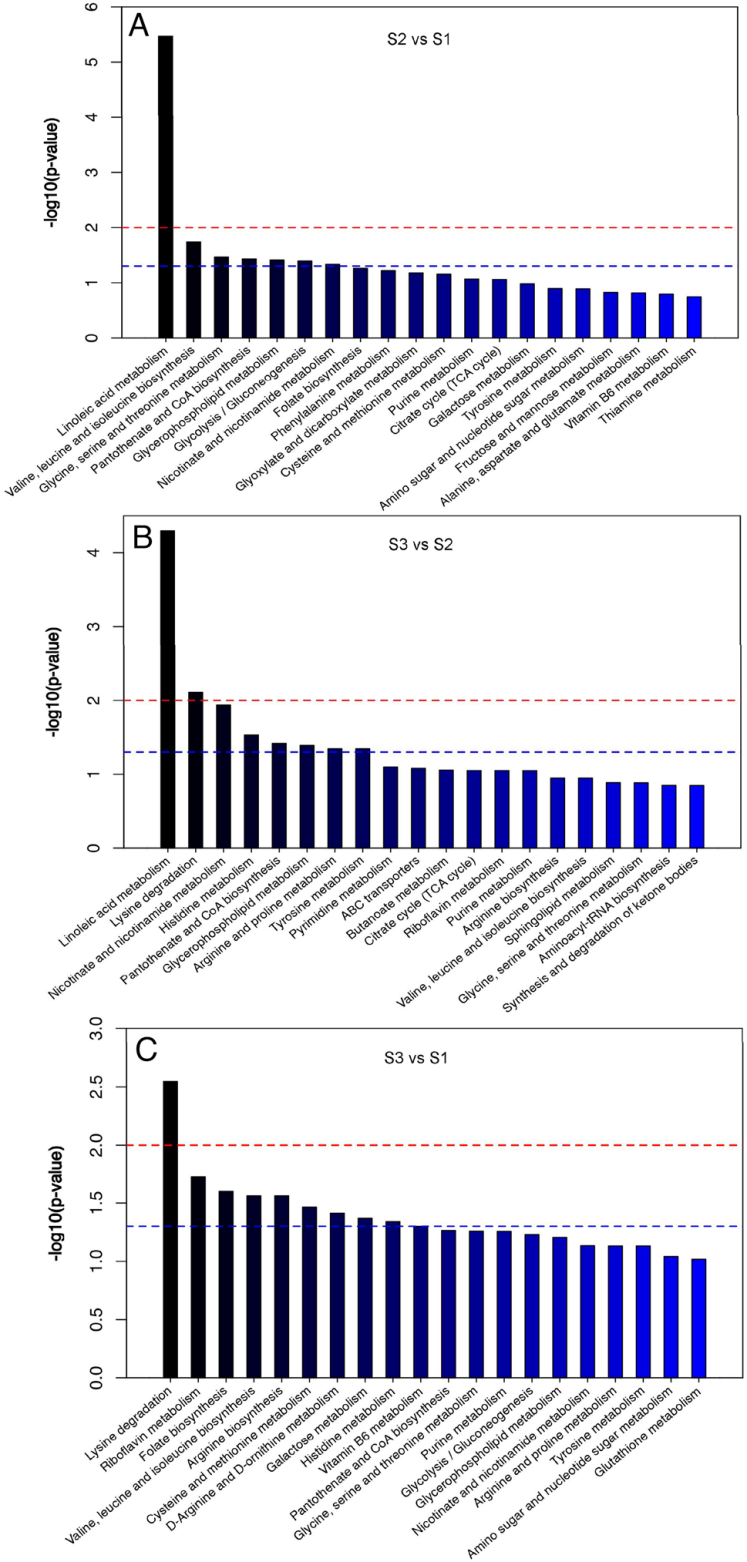
TOP-20 enrichment diagram of metabolic pathways in three comparable groups. (**A**) S2 vs S1, (**B**) S3 vs S2, and (**C**) S3 vs S1 group. *p*-value in metabolic pathway was the significance of enrichment of this metabolic pathway. The red line indicated that the *p* value was 0.01, and the blue line indicated that the *p* value was 0.05. When the top of the bar was higher than the blue line, the signal pathway represented that it was significant.

In this study, the metabolome analysis of different samples were identified with LC–MS. The differentially accumulated metabolites (DAMs) in S1, S2, and S3 groups were screened using |log_2_ foldchange| ≥ 1 and *p*-value < 0.05. The results of PCA showed that the metabolites were clearly separated among three samples group (Fig. 2A). Differential metabolites were detected in samples at different stages as shown in Fig. 2. Based on the criteria of an OPLS-DA model (VIP > 2.5 and *p*-value < 0.05), 514, 446, and 558 DEMs were detected in three comparable groups (S2 vs S1, S3 vs S2, and S3 vs S1), respectively. The DAMs of S3 were identified to be more abundant than S1 and S2, indicating that the DAMs change mainly happened in S3. Subsequently, 81, 76 and 93 DAMs were enriched into differential metabolites pathways mapped to the KEGG database, mainly including carbohydrate, amino acids, organic acids and secondary metabolites, of which metabolites involved in the metabolism of polysaccharides and lipid were more obviously accumulated, including ‘galactose metabolism’, ‘fructose and mannose metabolism’, ‘glycolysis’, ‘linoleic acid metabolism’ and ‘glycerophospholipid metabolism (Fig. 3; Suppl. Tables S1–S3).

**Figure 4:**
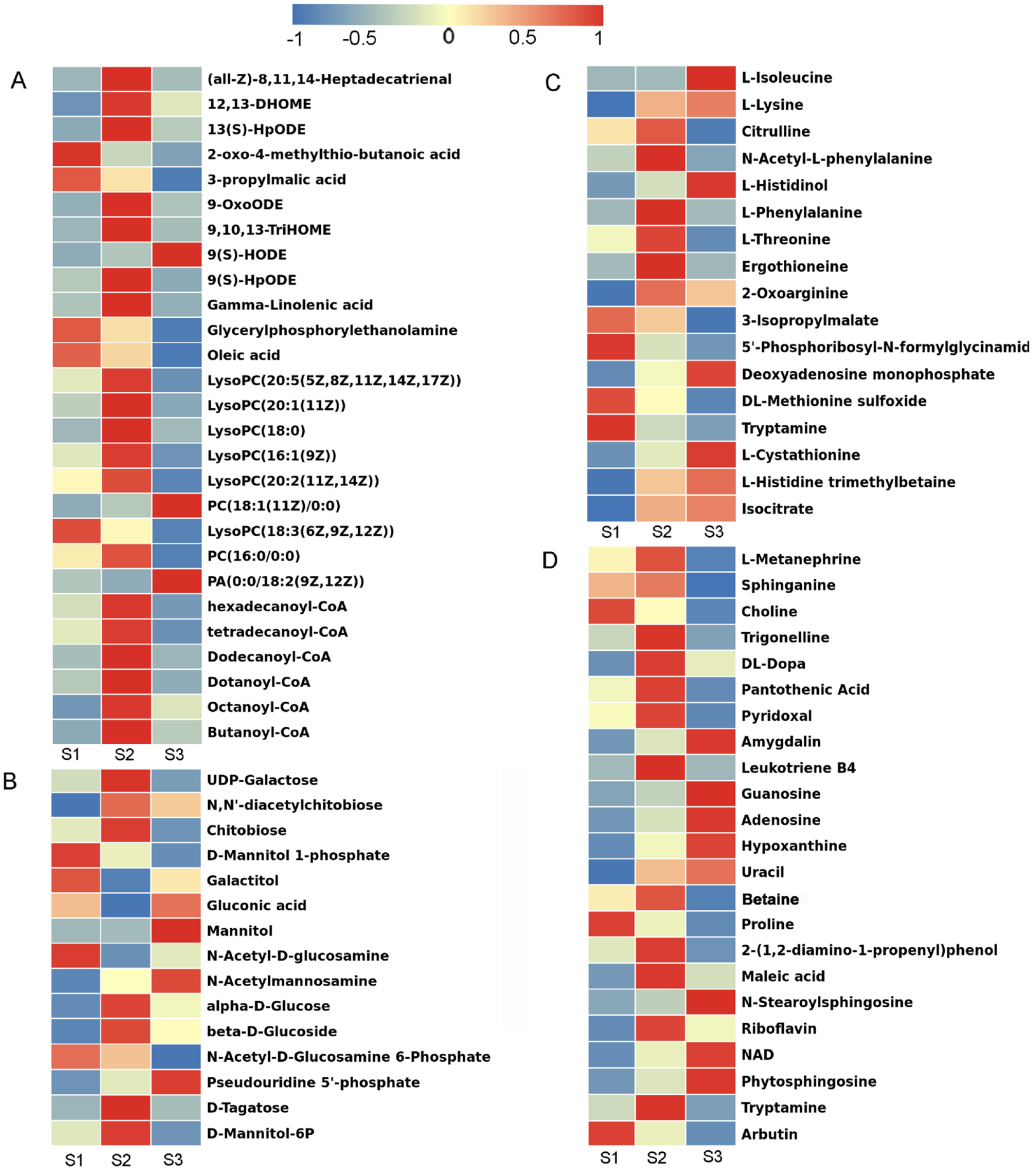
Heatmap of different type DAMs metabolites before and after fruiting body initiation with TBtools software. (**A**) Lipid metabolism. (**B**) Carbohydrate metabolism. (**C**) Amino acid metabolism. (**D**) Neurotransmitter, active factor. DAMs are selected based on VIP > 1 and *p* < 0.05 in any of the comparison groups.

As reported recently, primary and secondary metabolites such as sugars, bioactive substance were vital components that determined the initiation of fruiting body^1,4^. To further explore the differentially abundant metabolites in different groups, hierarchical clustering was applied to select DEMs and the change patterns of which were displayed in the heatmap (Fig. 4). Based on the top 20 annotated metabolic pathways, we further explored the fruiting body initiation related metabolites, including fatty acids, amino acids, sugars and activity factors that regulated the fruiting body initiation. Their accumulation patterns at different stages were shown in Fig. 4. Nineteen metabolites of the linoleic acid metabolism pathway were identified, among which seven metabolites (12,13-DHOME, 9(S)-HpODE, Gamma-Linolenic acid, 13(S)-HpODE, 9-OxoODE, 8,11,14-Heptadecatrienal and 9,10,13-TriHOME) showed high accumulation at primordium germination stages. Meanwhile, six metabolites of the fatty acid metabolism pathway were identified, all of which were also showed much more enriched at primordium germination stages (Fig. 4A). Half of sugars such as α-d-Glucose, d-Mannitol-6P and β-d-Glucosidetagatose were also obviously accumulated at this period (Fig. 4B). Eighteen metabolites of amino acid metabolism were detected, most of them were markedly accumulated at primordium germination period, like l-lysine, l-threonine (Fig. 4C). More interestingly, some active substance such as dl-Dopa, arbutin, trigonelline and maleic acid peaked at primordium stages followed by a gradual decrease (Fig. 4D), which might play vital role in initiation process of fruiting body.

### Transcriptome analyses of Chinese cordyceps mycelium related to fruiting body initiation

**Figure 5:**
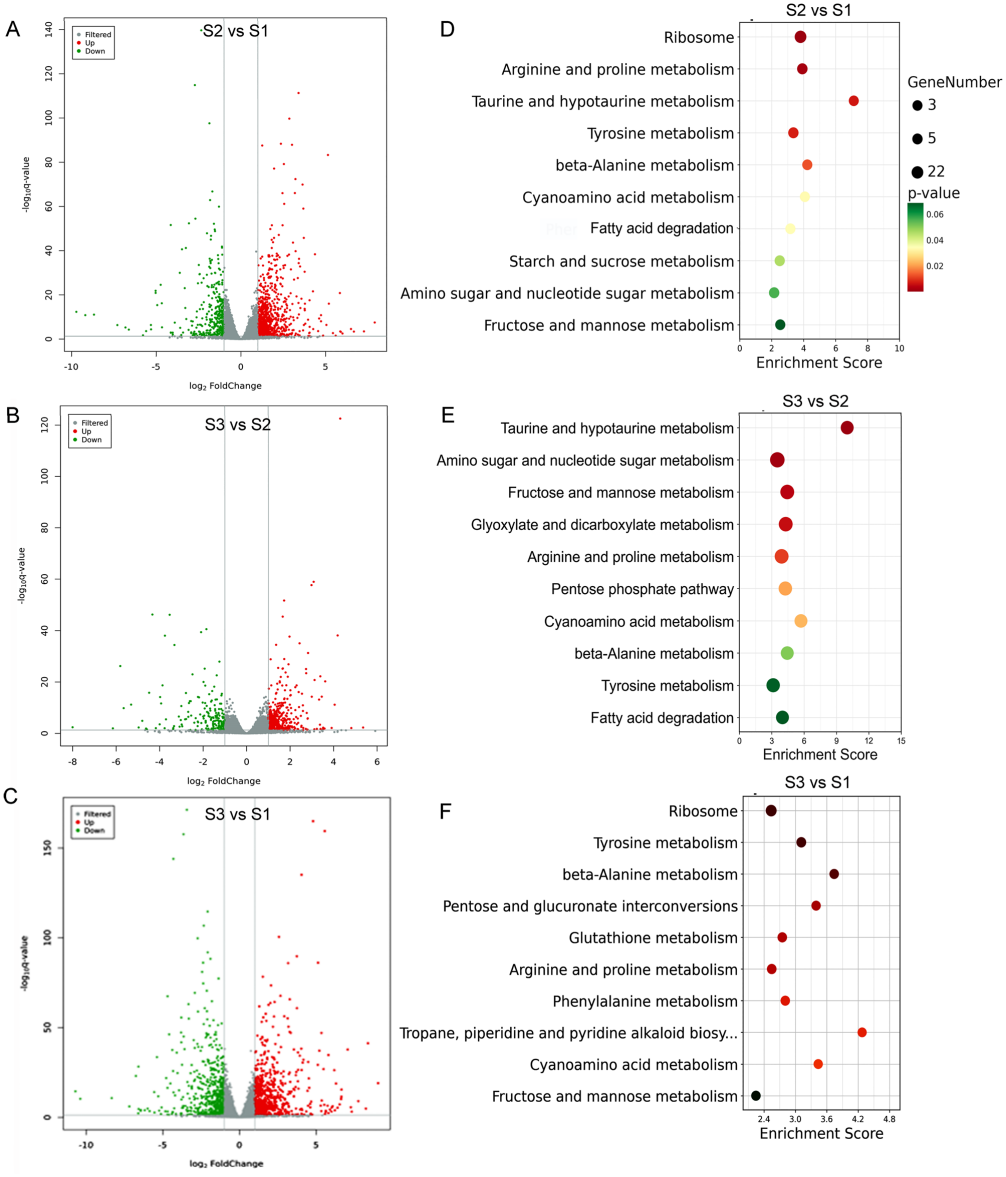
Bar plot showing numbers of DEGs in S2 vs S1 (**A**), S3 vs S2 (**B**), and S3 vs S1 (**C**) groups. Differential genes with *p* value < 0.05 and FC value > 2 were selected for KEGG enrichment pathways. Top 10 pathways as enrichment factor from DEGs in S2 vs S1 (**D**), S3 vs S2 (**E**), and S3 vs S1 (**F**) groups. The horizontal axis represents the differential expression multiple, and the vertical axis represents the degree of difference in gene meaning. KEGG enrichment pathways were created in http://www.kegg.jp/kegg/kegg1.html.

A transcriptomic comparison was conducted to identify differentially expressed genes (DEGs) in S2 vs S1, S3 vs S2, and S3 vs S1 comparative groups, respectively. Transcriptomic analysis showed that a total of 3157 DEGs were identified, among which 1406 DEGs were identified in S2 vs S1 group, including 768 upregulated and 638 downregulated genes (Fig. 5A). There were 1174 DEGs in S3 vs S2 group, containing 812 upregulated and 362 downregulated genes (Fig. 5B). The remaining DEGs were in S3 vs S1 group, involving 389 upregulated and 188 downregulated genes (Fig. 5C), suggesting that a considerable proportion of the transcriptomic changes occurred during the primordium initiation process.

Based on the statistical significance criterion for multiple testing correction (adjusted *p*-value), the KEGG terms of DEGs in S2 vs S1 groups were mainly enriched in ribosomes, amino acid metabolic pathways (arginine and proline, tyrosine, alanine and phenylalanine) and starch and sucrose, fructose and mannose metabolism (Fig. 5D). The significantly enriched KEGG terms of DEGs in S3 vs S2 were tryptophan, hypotaurine, nucleotide sugar, fructose and mannose, glyoxylate, arginine and proline metabolic pathways (Fig. 5E). The top enriched KEGG terms of DEGs in S3 vs S1 group were ribosomes, tyrosine, β-alanine, arginine, proline, pentose glucuronate, hypotaurine and glutathione metabolic pathways (Fig. 5F). Collectively, the above mentioned KEGG analysis suggested that DEGs were mainly involved in ribosomes, carbohydrate, fatty acid degradation and amino acid metabolism during the process of primordium initiation.

### Combined metabolomic and transcriptomic analyses in Chinese cordyceps before and after fruiting body initiation

**Figure 6:**
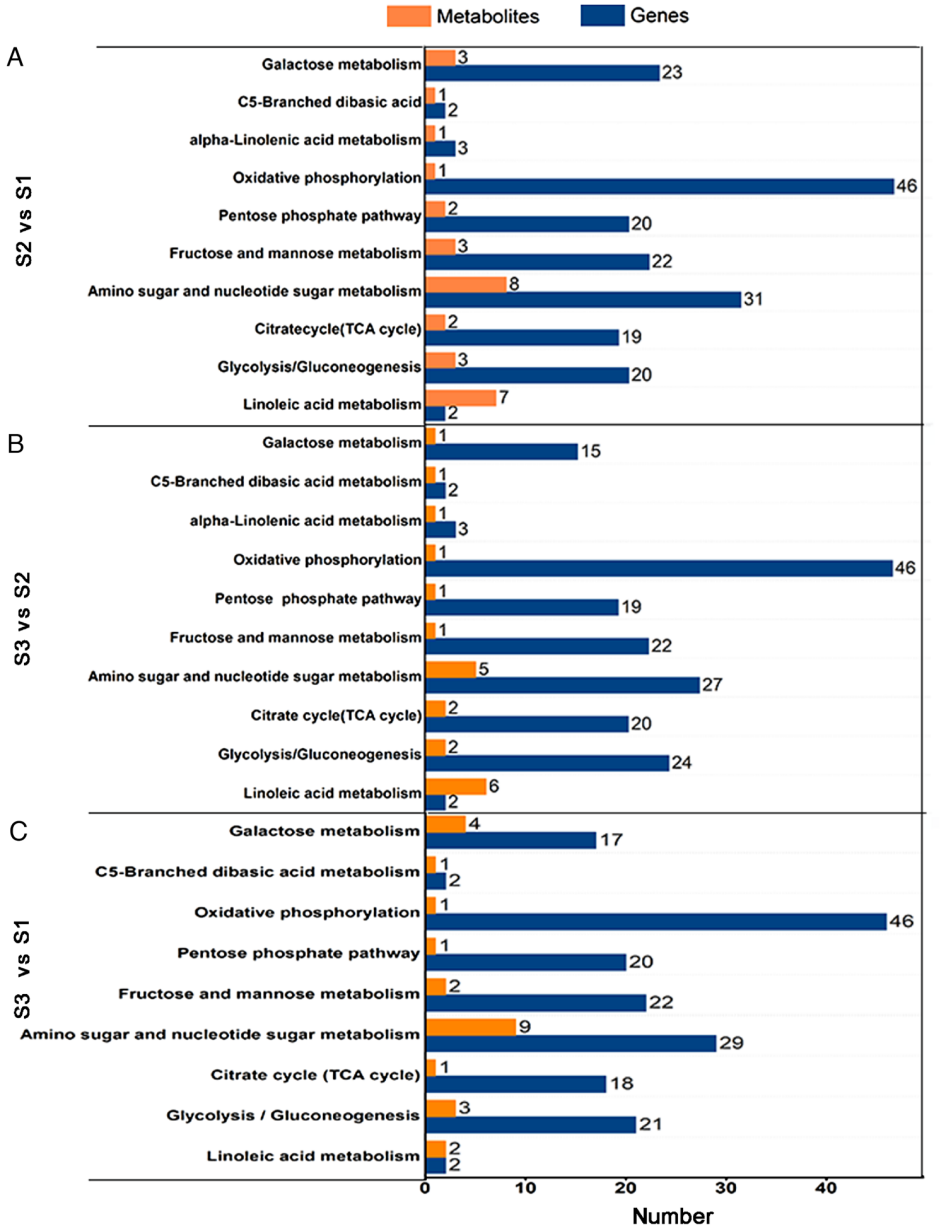
Integrated analyses of the transcriptome and metabolome of the KEGG pathways related to sugars and lipid. As shown in Suppl. Table S4. Three comparison groups of S2 vs S1 (**A**), S3 vs S2 (**B**), and S3 vs S1 (**C**) were selected. The specific name of the KEGG pathway was provided on the left of the bar chart, and the numbers of relative DEGs and DAMs were on the right of the bar. KEGG enrichment pathways were created in http://www.kegg.jp/kegg/kegg1.html.

To further explore the relationship between gene expression and metabolite accumulation in Chinese cordyceps, conjoint biological annotations was conducted. Based on the combination of transcriptome and metabolome data, the common changed KEGG pathways relevant to carbohydrate and lipid metabolism, the DEGs and DAMs between comparable group were identified (Fig. 6; Suppl. Table S4). In these comparison groups (S2 vs. S1, S3 vs. S2, and S3 vs. S1), ‘oxidative phosphorylation’, ‘amino sugar and nucleotide sugar metabolism’ contained more DEGs, while ‘linoleic acid metabolism’ (S2 vs. S1 and S3 vs. S2) and lysine metabolism (S3 vs S1) were the second most enriched pathways, there were 24, 30, and 18 DAMs connected with carbohydrate and lipid metabolism were found in the common KEGG pathway analysis of three comparison groups.

**Figure 7:**
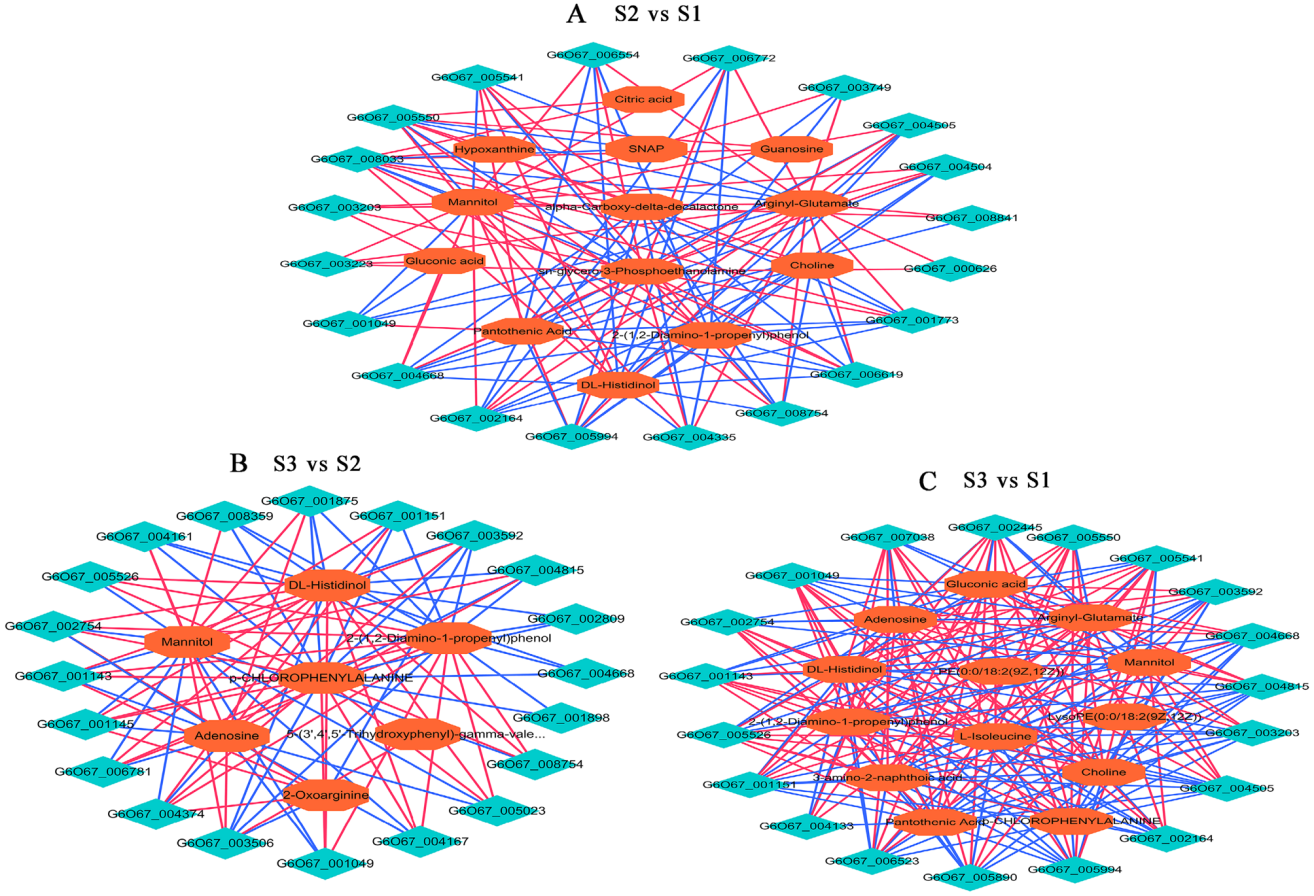
Correlation network of DEGs and DAMs involved in sugars and lipid metabolism. (**A**) S2 vs S1, (**B**) S3 vs S2, (**C**) S3 vs S1. Green circles indicate genes, and orange circles indicate metabolites. Lines colored in “red” and “green” represent positive and negative correlations, respectively, as determined by a Pearson's correlation coefficient > 0.8 or < − 0.8 (q-value < 0.1), respectively.

The correlations between the top 20 DEGs and DAMs were conducted with “Pearson’s algorithm”. The network analysis intuitively illustrated DEGs and DAMs related to sugar and lipid metabolism in three comparison groups (Fig. 7). In S2 vs S1 group, some glycerophospholipid metabolism related DAMs like sn-glycero-3-phosphoethanolamine (PE) and choline were highly accumulated and positively regulated by many DEGs as genes encoding hypothetical protein (KAF4507556.1), glutathione-dependent formaldehyde -activating enzyme (EQL01886), linoleate diol synthase (EQK99321.1), while these DAMs were negatively correlated with gene encoding vip1 (EQL00872.1). However, this DEG positively regulated pantothenic acid accumulation. Some organic acids DAMs related to carbohydrate acid metabolism, such as gluconic acid and citric acid were also obviously enriched and positively regulated by gene coding cell wall protein (KAF4597257.1) and phosphatidyl inositol-specific phospholipase C (KAF026501.1). These two organic acids were closely connected with carbohydrate metabolism. In addition, transmembrane protein (KAF4582033.1) was positively correlated with mannitol (r = 0.97) and dl-histidinol (r = 0.96). In S3 vs S2 group, The DAMs, such as 2-(1,2-diamino-1-propenyl) phenol and mannitol were still highly accumulated and positively regulated by another two genes encoding hypothetical protein (EQK99003.1) and PAN domain containing protein (EQL03645.1), while these two DEGs were negatively connected with accumulation. Collectively, compared with pre-primordium period, the DAMs related carbohydrate acid metabolism (gluconic acid and citric acid), glycerophospholipid metabolism (PE, LysoPE, choline), defensive substance [2-(1,2-diamino-1-propenyl) phenol] and mannitol were markedly enriched at after-primordium period.

### Candidate genes related to the carbohydrate metabolism and fatty acid degradation at initiation stage of fruiting body

**Figure 8:**
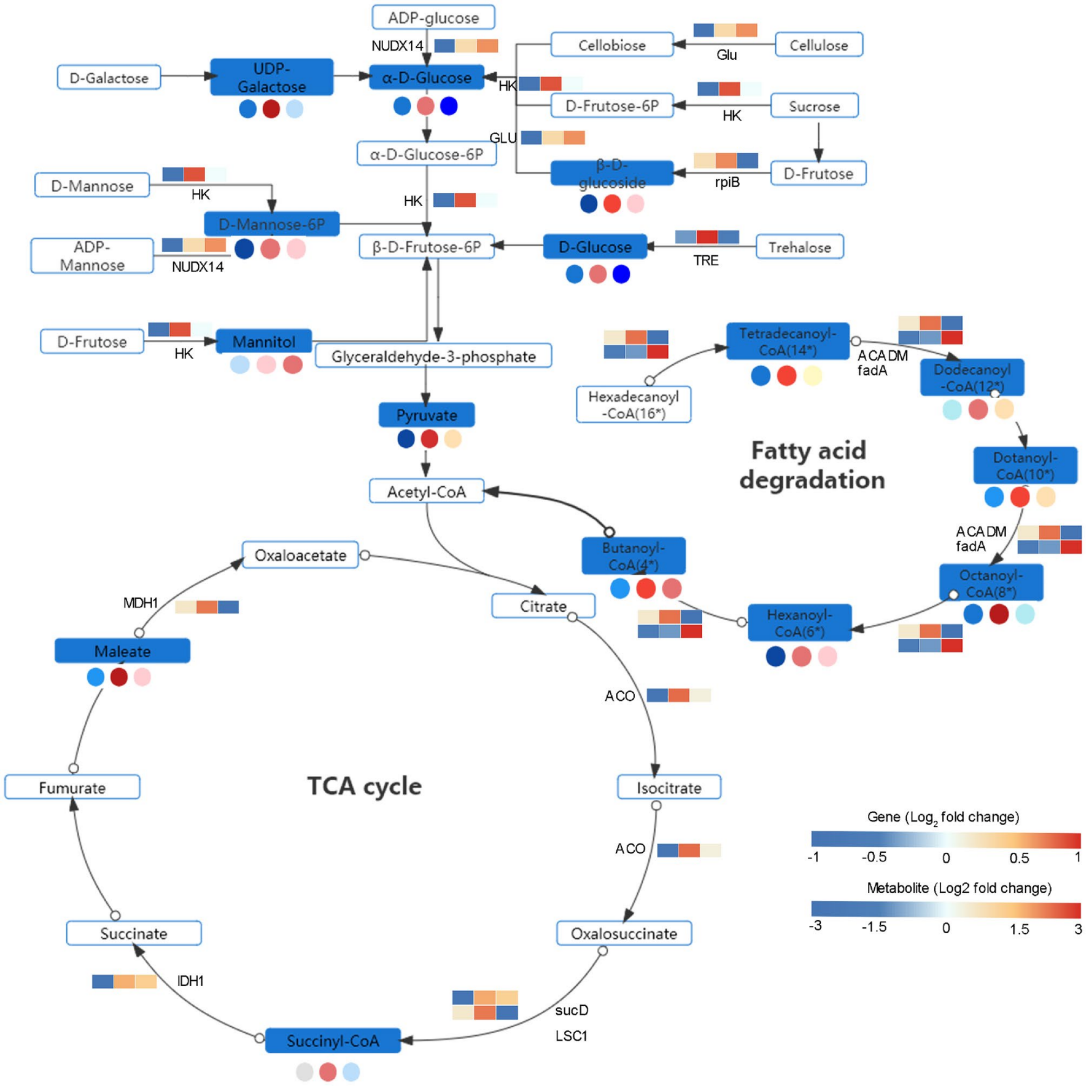
The DEGs and DEMs involved in the carbohydrate metabolism and fatty acid degradation during the initiation process of fruiting body. The blue rectangle represented the significantly accumulated metabolites. The rectangle was divided into three equal parts (the left of the rectangle represents DEGs in S1, the middle represented DEGs in S2, and the right represented DEGs in S3. The color in the rectangle. The circle represented DEMs, three equal parts was similar to DEGs. *HK* hexokinase, *NUDX14* ADP-sugar diphosphatase, *rpiB* ribose 5-phosphate isomerase B, *TRE* trehalose phosphate synthase, *Glu* beta-glucosidase, *ACADM* acyl-CoA dehydrogenase, *fadA* acetyl-CoA acyltransferase, *MDH1* malate dehydrogenase, *sucD* succinyl-CoA synthetase, *ST* succinate thiokinase, *ACO* aconitase, *IDP1*:isocitrate dehydrogenase.

Based on the integrated analysis of genes and metabolites, energy supply for fruiting body initiation from varied sugar metabolism and fatty acid degradation was speculated as shown in Fig. 8. In carbohydrate metabolism, a hexokinase (*HK*) gene showed higher expression at S2 stage than that in S1 stage in several pathways. An ADP-sugar diphosphatase (*NUDX14*) gene exhibited an increasing tendency and then maintained a steady level at S3 stage. A ribose 5-phosphate isomerase B (*rpiB*) and trehalose phosphate synthase (*TRE*) were all significantly upexpressed at S2 stage. Beta-glucosidase (*Glu*) showed a continuing increasing-tendency after fruiting body initiation (S2). Compared with S1, the content of downstream metabolite pyruvate was markedly increased by 5 times at S2 stage. These results indicated that *HK*, *NUDX14*, *rpiB*, *TRE* and *Glu* were key genes in carbohydrate metabolism at initiation stage of fruiting body, which strongly regulated the synthesis of key metabolites, especially pyruvate. In fatty acid degradation pathways, two key genes, acyl-CoA dehydrogenase (*ACADM*) and acetyl-CoA acyltransferase (*fadA*), were highly expressed in almost every step of fatty acid degradation at S2 stage, which significantly regulated the metabolites enrichment of every steps such as hexadecanoyl-CoA, tetradecanoyl-CoA, lauroyl-CoA. In the tricarboxylic acid cycle, malate dehydrogenase (*MDH1*), two succinyl-CoA synthetase (*sucD* and *IDP1*) were highly expressed at S2 stage, and the related metabolites, malate and succlnyl CoA, were showed strongly accumulation. The above results suggested fatty acid degradation (β-oxidation) was the most important way providing a large amount of energy for organisms by coupling with TCA at fruiting body initiation period.

### Candidate genes related to the glycerophospholipid and linoleic acid metabolism at initiation stage of fruiting body

**Figure 9:**
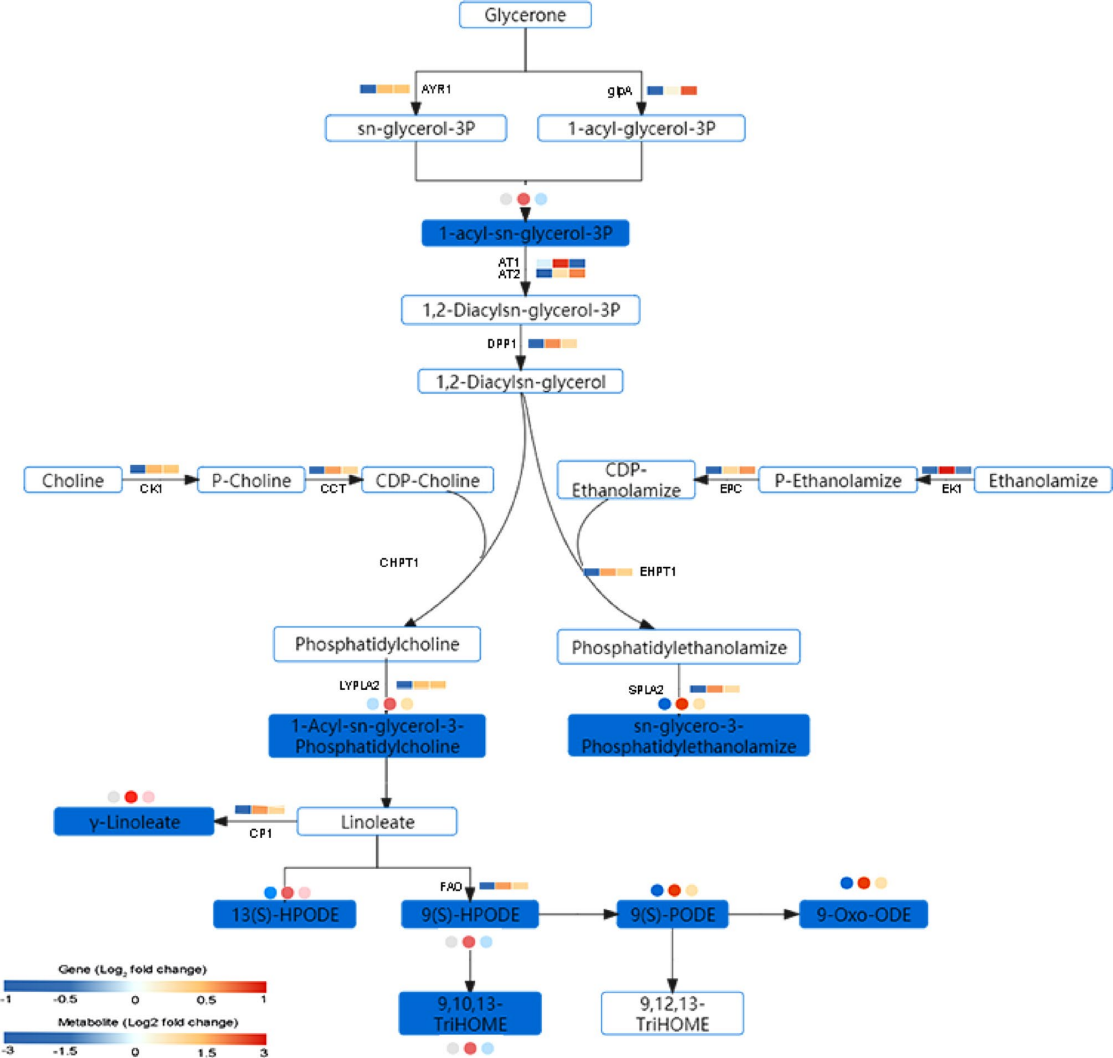
The DEGs and DEMs involved in the glycerophospholipid and linoleic acid metabolism during the initiation process of fruiting body. The blue rectangle represents the significantly accumulated metabolites. The rectangle was divided into three equal parts (the left of the rectangle represents DEGs in S1, the middle represented DEGs in S2, and the right represented DEGs in S3. The circle represented DEMs, three equal parts was similar to DEGs. *glpA* glycerol-3-phosphate dehydrogenase, *AYR1* 1-acylglycerone phosphate reductase, *DPP1* diacylglycerol diphosphate phosphatase, *AT2* acyltransferase, *CK* choline kinase, *CCT* cytidylyltransferase, *EPC* ethanolamine-phosphate cytidylyltransferase, *AT1* acyltransferase1, *EK* ethanolamine kinase, *CHPT1* CDP choline transferase, *EHPT1* CDP ethanolamine transferase, *LYPLA2* cytosolic phospholipase A2, *SPLA2* ethanolaminephosphotransferase, *CP1* cytosolic phospholipase, *FAO* fatty acid oxygenase.

Based on the integrated analysis of genes and metabolites, the glycerophospholipid and linoleic acid metabolism of Chinese cordyceps was speculated as shown in Fig. 9. The expression change of protease genes and metabolites from S1 to S3 were displayed. The 12 DEGs were found to be related to glycerophospholipid metabolism. Of them, glycerol-3-phosphate dehydrogenase (*glpA*), 1-acylglycerone phosphate reductase (*AYR1*), diacylglycerol diphosphate phosphatase (*DPP1*), acyltransferase (*AT2*), choline kinase (*CK*), cytidylyltransferase (*CCT*) and ethanolamine-phosphate cytidylyltransferase (*EPC*) were markedly upregulated at S2 stage than that in S1, and then showed consistently increasing tendency at S3 stage rather than acyltransferase1 (*AT1*) and ethanolamine kinase (*EK*). These DEGs obviously manipulated the synthesis of key metabolites, especially in 1,2-Diacyl-sn-glycero-3-phosphocholine (lecithin) and 1-Acyl-2-acyl-sn-glycero-3- phosphoethanolamine (cephalin). All of which were considered to be the superior neuronutrients, essential for fruiting body initiation. Lecithin was further decomposed into linoleic acid and other metabolites (9(S)-HPODE, 13(S)-HPODE), which was strongly regulated by cytosolic phospholipase A2 zeta (*CPA2*) and fatty acid oxygenase (*FAO*). Linoleic acid and these metabolites might be essential for fruiting body initiation.

### Validation of candidate DEGs by quantitative real-time PCR

**Figure 10:**
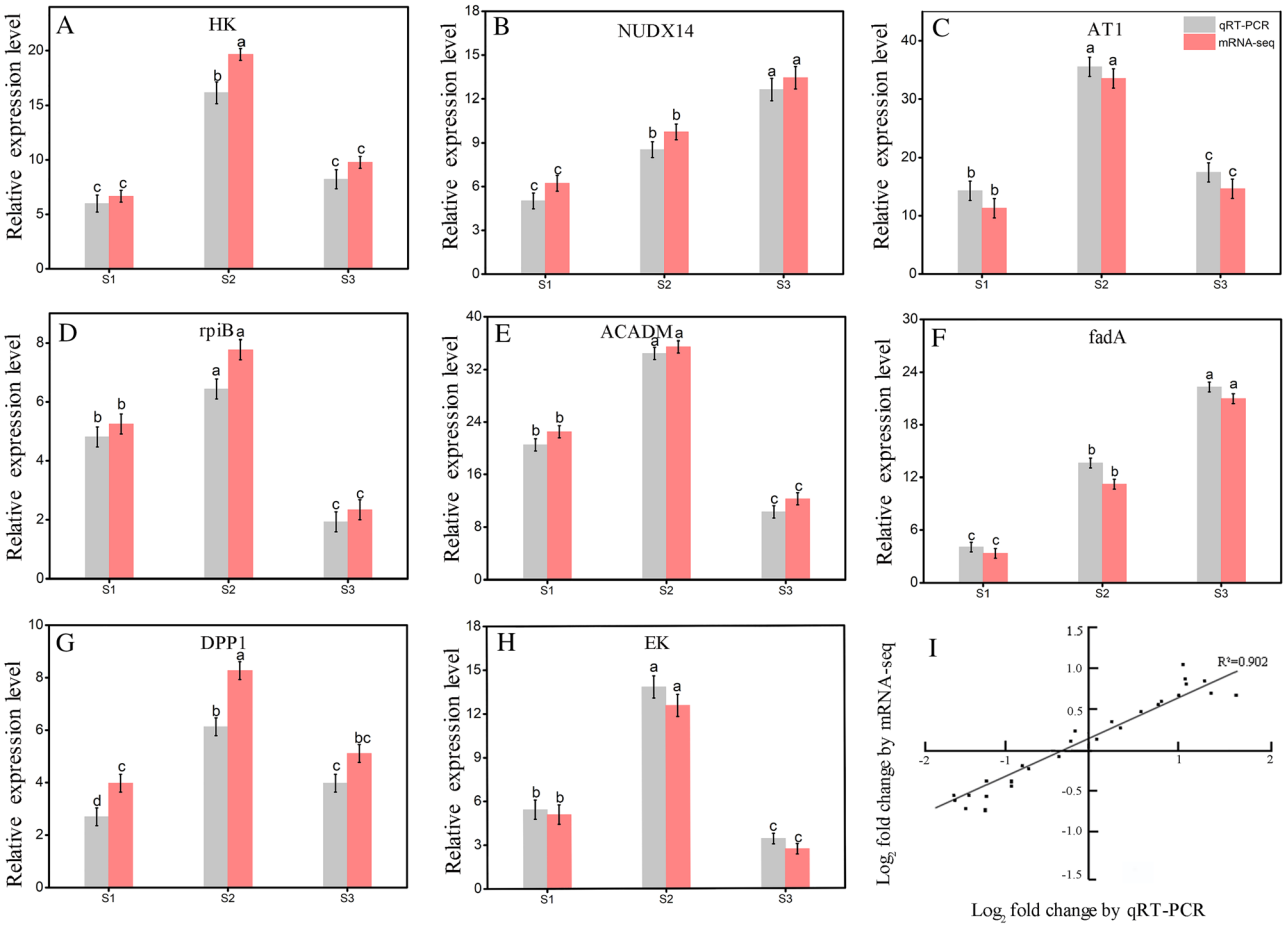
Expression pattern validation (**A**–**H**) and linear dependence relation between the log_2_ values of the key gene expression ratios obtained from RNA-seq and qRT-PCR (**I**).

To further verify the accuracy of RNA-seq analysis data, eight DEGs (*HK,* *NUDX14,* *rpiB,* *ACADM,* *fadA,* *AT1,* *DPP1,* *EK*) were randomly selected for qRT-PCR verification. Figure 10 showed the patterns of RNA-Seq and RT-PCR expressions on these genes of 8 DEGs. The result of two techniques were consistent, a correlation coefficient (R2) of 90.20%, indicating the high-throughput transcriptome sequencing was reliable.

## Discussion

The initiation and formation of fruit body of Chinese cordyceps was the key node in the growth process. The research on the mechanism of changes in this growth stage was the basis for the industrialization of artificial cultivation of Chinese cordyceps. Some metabolites have been proved to be closely involved in the formation of fruiting bodies in fungus. Such as polysaccharides, nucleosides, urea, sterols, mannitol, polyphenol and others. Some previous studies found that polysaccharides were mainly concentrated in the area of cap and stalk, and decreased after it developed into fruiting bodies in oyster mushroom^30,31^. The findings of our study were similar to those observations, that is, the polysaccharide content in S2 was higher than that in S1 and S3 (Fig. 1), indicating that subsequent stage of fruiting body development was the sexually developed ascus shell and spore, which were the most critical organs for fungi to reproduce offspring and must give priority to nutrient supply and growth. In fungus, organisms could produce and accumulate mannitol under osmotic stress to improve osmotic pressure tolerance^32^. Qian et al. found that the mannitol content was the highest before primordium matured compared different growth stages in *Cordyceps* *sinensis*^33^. Our results was consistent with this study, the mannitol content showed a trend of continuous increase and peaked at S2 stage. Wyatt et al. found that the biosynthesis of mannitol in *N.* *focheri* mainly depended on 1-phosphate mannitol dehydrogenase (*mpdA*). When *mpdA* was absent, the ascospores of *N.* *focheri* couldn’t develop completely, indicating that mannitol played an important role in the sexual development and ascospore formation in *N.* *focheri*^34^. Polyphenols was the important defense substances in organism secondary metabolism^35^. Previous studies have detected it in laboratory cultured conditions and peaked in mycelium in Chinese cordyceps^36^. Similarly, in our study, polyphenols content continuously increased and peaked at S1 stage (Fig. 1), and this was further confirmed by metabonomics results (Fig. 7). Some studies have demonstrated that phenols in Chinese cordyceps have strong antioxidant and anti-cytotoxic potential during its development process^37,38^. So we inferred it might be as antioxidant and immunoactivator to enhance antioxidant capacity and immune-stimulating activities after primordium formated in Chinese cordyceps.

Carbohydrate metabolism and fatty acid degradation were essential for fruiting body initiation and formation to provide nutrients and energy^35^. In the present study, UDP-Galactose, α-d-Glucose, d-Mannitol-6P and β-d-Glucoside obviously increased in S2 and decreased in S3. Compared to these sugars, mannitol showed continuously increasing trend and peaked at S3. Among which UDP-Galactose and β-d-Glucoside converted into α-d-Glucose, d-Mannitol-6P and mannitol converted into β-d-Fructose, then they converted to pyruvate, and these accumulated sugars were mainly positively regulated by *HK*, *NUDX14*, *rpiB* and *TRE*, especially in *HK,* it played crucial role in various sugar metabolisms*,* which was consistent with the results obtained in previous study that *HK* as key gene involved in mannose or polysaccharide biosynthesis in *O.* *sinensis*^37^*.* The rapid accumulation of these sugars in the medium at S2 stage might afford enough nutrients and energy by glycolytic pathway. The similar accumulation pattern of sugar has been also reported in other fungus^38,39^. However, the pyruvate produced only by glycolysis pathway was insufficient to supply the capacity for fruiting bodies initiation to initiate into the TCA cycle. Therefore, we speculated fruit body initiated the fatty acid degradation pathway to assist the glycolysis pathway to increase capacity. β-oxidation pathway has been proved to be the crucial route of production capacity for the formation of sclerotia and fruiting bodies of *O.* *sinensi*s^29^. Our result was consistent with this study, showing that metabolites related to β-oxidation pathway of palmitic acid were all markedly accumulated at S2 stage, which were obviously positively regulated by *ACADM* and *fadA.* It was likely that the glycolysis and β-oxidation pathway of palmitic acid pathways worked together to produce large amounts of acetyl-coA and then entered into the tricarboxylic acid cycle (TCA) pathway to produce large amounts of ATP for fruiting body initiation.

It has been demonstrated that phospholipid metabolism regulated lipid, lipoprotein and whole-body energy metabolism in numerous dietary studies^1,40^, and even activated the transmitter and increased the speed of brain link information transmission^41^. Phosphatidylcholine (PC) and phosphatidylethanolamine (PE) were the most abundant phospholipids in all mammalian cell membranes^42^. Similarly, in our study, we detected both excretion of PC and PE were all markedly accumulated at S2 stage, which were positively direct-regulated by lysophospholipase (*LYPLA2*) and ethanolamine kinase (*EHPT1*). Linoleic acid [(9Z,12Z)-9,12-octadecadienoic acid], the most abundant fatty acid in PC, was an essential fatty acid for most animals, creating proper fluidity and permeability in cell membranes^43,44^. Similarly, in our metabolomic observations, ‘linoleic acid metabolism’ was the most enriched pathway (Fig. 3), seven metabolites(12,13-DHOME, 9(S)-HpODE, Gamma-Linolenic acid, 13(S)-HPODE, 9-OxoODE, 8,11,14-Heptadecatrienal and 9,10,13-TriHOME) showed high accumulation at S2 stages (Fig. 9), it might play critical role in creating proper fluidity and permeability in cell membranes collaborated with other osmotic adjustment substances. Furthermore, 13(S)-HPODE of oxidized linoleic acid metabolites (OXLAMs) has been reported to regulate the neural development of young mice^45,46^. Thus, we speculated it might play a similar role in the initiation of fruiting bodies.

In the future, firstly, we will added some preferred energy-metabolizing substances found in our observation which trigger the develop of pins or fruit body to modify the original culture medium for more efficient production of the fungus. Secondly, after modified the medium components, the culture conditions were further optimized to be better benefit for production of the fungus. Thirdly, many industrially produced macro-fungus have confirmed that the breeding of high quality strains was the most effective means to optimize strain production, so we consider to breed high quality strains based on above-optimized culture medium later.

## Conclusion

In this study, combined analysis of transcriptome and metabonomics was conducted to reveal the energy supply mechanism involved in fruiting body initiation in Chinese cordyceps. Metabolite and gene expression analyses indicated that metabolites and genes related to starch, sucrose, fructose, mannose, linoleic acid, fatty acids degradation and glycerophospholipid metabolism played critical roles in fruiting body initiation in Chinese cordyceps. All of these metabolic pathways might work cooperatively to generate enough acyl-CoA, and then enter into TCA cycle to provide energy for fruiting body initiation. Our finding provided important information for further exploring the energy metabolic mechanisms of realizing the industrialization of Chinese cordyceps artificial cultivation.

## Supplementary Information

Supplementary Table S1.

Supplementary Table S2.

Supplementary Table S3.

Supplementary Table S4.

## Data Availability

The datasets generated and analysed during the current study are available in SRA database (https://www.ncbi.nlm.nih.gov/sra/PRJNA939521).
