# Supplementary material for: Transcriptome and metabonomics combined analysis revealed the energy supply mechanism involved in fruiting body initiation in Chinese cordyceps
Source: Sci Rep. 2023 Jun 12;13:9500. doi: 10.1038/s41598-023-36261-7 (PMC10261108; doi:10.1038/s41598-023-36261-7)
Supplement: Supplementary file 4 — Supplementary Table S4. [file 41598_2023_36261_MOESM4_ESM.doc]

| groups | Metabolic pathways | gene | metabolite |
| --- | --- | --- | --- |
| B vs A | Linoleic acid metabolism | 2 | 7 |
|  | Glycolysis / Gluconeogenesis | 20 | 3 |
|  | Citrate cycle (TCA cycle) | 19 | 2 |
|  | Amino sugar and nucleotide sugar metabolism | 31 | 8 |
|  | Fructose and mannose metabolism | 22 | 3 |
|  | Pentose phosphate pathway | 20 | 2 |
|  | Oxidative phosphorylation | 46 | 1 |
|  | alpha-Linolenic acid metabolism | 3 | 1 |
|  | C5-Branched dibasic acid metabolism | 2 | 1 |
|  | Galactose metabolism | 23 | 3 |
| A vs C | Linoleic acid metabolism | 2 | 2 |
|  | Glycolysis / Gluconeogenesis | 21 | 3 |
|  | Citrate cycle (TCA cycle) | 18 | 1 |
|  | Amino sugar and nucleotide sugar metabolism | 29 | 9 |
|  | Fructose and mannose metabolism | 22 | 2 |
|  | Pentose phosphate pathway | 20 | 1 |
|  | Oxidative phosphorylation | 46 | 1 |
|  | alpha-Linolenic acid metabolism | 3 | 1 |
|  | C5-Branched dibasic acid metabolism | 2 | 1 |
|  | Galactose metabolism | 17 | 4 |
| B vs C | Linoleic acid metabolism | 2 | 6 |
|  | Glycolysis / Gluconeogenesis | 24 | 2 |
|  | Citrate cycle (TCA cycle) | 20 | 2 |
|  | Amino sugar and nucleotide sugar metabolism | 27 | 5 |
|  | Fructose and mannose metabolism | 22 | 1 |
|  | Pentose phosphate pathway | 19 | 1 |
|  | Oxidative phosphorylation | 46 | 1 |
|  | alpha-Linolenic acid metabolism | 3 | 1 |
|  | C5-Branched dibasic acid metabolism | 2 | 1 |
|  | Galactose metabolism | 15 | 1 |

**Suppl. Table S4**. KEGG pathways of transcriptome and metabolome related to sugars and lipid
